# CIRS: A Multi-Agent Machine Learning Framework for Real-Time Accident Detection and Emergency Response

**DOI:** 10.3390/s25185845

**Published:** 2025-09-19

**Authors:** Sadaf Ayesha, Aqsa Aslam, Muhammad Hassan Zaheer, Muhammad Burhan Khan

**Affiliations:** 1 Department of Electrical Engineering, National University of Computer and Emerging Sciences, Karachi 75030, Pakistan; 2 Department of Computer Science, National University of Computer and Emerging Sciences, Karachi 75030, Pakistan; aqsa.aslam@nu.edu.pk (A.A.); mhassanzaheer@gmail.com (M.H.Z.)

**Keywords:** multi-agent systems, accident detection, YOLOv11, object classification, vision-language models, emergency response systems, traffic incident management

## Abstract

Road traffic accidents remain a leading cause of fatalities worldwide, and the consequences are considerably worsened by delayed detection and emergency response. Although several machine learning-based approaches have been proposed, accident detection systems are not widely deployed, and most existing solutions fail to handle the growing complexity of modern traffic environments. This study introduces Collaborative Intelligence for Road Safety (CIRS), a novel, multi-agent, machine-learning-based framework designed for real-time accident detection, semantic scene understanding, and coordinated emergency response. Each agent in CIRS is designed for a distinct role perception, classification, description, localization, and decision-making, working collaboratively to enhance situational awareness and response efficiency. These agents integrate advanced models: YOLOv11 for high-accuracy accident detection and VideoLLaMA3 for contextual-rich scene description. CIRS bridges the gap between low-level visual analysis and high-level situational awareness. Extensive evaluation on a custom dataset comprising (5200 accident, 4800 nonaccident) frames demonstrates the effectiveness of the proposed approach. YOLOv11 achieves a top-1 accuracy of 86.5% and a perfect top-5 accuracy of 100%, ensuring reliable real-time detection. VideoLLaMA3 outperforms other vision-language models with superior factual accuracy and fewer hallucinations, generating strong results in the metrics of BLEU (0.0755), METEOR (0.2258), and ROUGE-L (0.3625). The decentralized multi-agent architecture of CIRS enables scalability, reduced latency, and the timely dispatch of emergency services while minimizing false positives.

## 1. Introduction

Road traffic accidents remain a leading cause of fatalities, with over 1.35 million lives lost annually worldwide [1]. As urban populations grow and road networks expand, the scale and complexity of traffic increase significantly, intensifying the need for fast, reliable accident detection. Every second counts: the earlier an incident is detected and emergency services are notified, the higher the chances of minimizing injuries and saving lives. However, current accident detection systems often fall short in delivering accurate and timely responses due to their reliance on static rule-based logic or single-model classifiers.

Despite the progress in intelligent transportation systems (ITS), challenges such as low-resolution CCTV footage, occlusions, varied weather conditions, and the dynamic nature of urban traffic continue to hinder detection accuracy and real-time responsiveness [2]. Traditional centralized systems lack the scalability and adaptability required for real-world deployments in high-density traffic environments [3]. Moreover, many existing solutions treat accident detection as an isolated task, overlooking the importance of context, reasoning, and collaboration among perception and decision-making modules. To address these limitations, this study introduces the Collaborative Intelligence for Road Safety (CIRS) framework, a novel, multi-agent, machine learning-based system that reconceptualizes accident detection as a decentralized, context-aware, and collaborative process. The framework is built upon the principles of multi-agent systems (MASs) come into play [4], offering a biologically inspired approach in which autonomous agents, much like ants coordinating to find food or birds flying in formation, collaborate to perform complex tasks efficiently and resiliently.

The CIRS framework uses a MAS to improve real-time accident detection and response via CCTV video streams. The MAS includes five agents: perception (video capture and preprocessing), classification (identify accident frames), VLM Description (summaries), communication (coordination), and decision-making (response and robot integration). CIRS functions in two stages: classification and captioning. For classification, YOLOv11 is used for binary accident detection, chosen for its accuracy, speed, and efficiency [5]. High precision minimizes false positives, and the redundancy of the video stream helps recover missed detections. In captioning, VideoLL3 generates natural language descriptions of accidents, detailing vehicle types and severity, improving decision-making [6]. The CIRS framework mainly highlights accident detection, which is key for captioning and severity evaluation. MAS performance is thoroughly assessed with quantitative metrics such as precision, recall, F1-score, qualitative results (captioned frames), and visual aids (heatmaps and attention maps), proving its real-world effectiveness.

The primary contributions of this research are as follows:

- A novel multi-agent framework, Collaborative Intelligence for Road Safety (CIRS), is introduced to reconceptualize accident detection as a decentralized, context-aware process, improving system scalability and resilience.
- Beyond binary accident detection, the framework integrates semantic vehicle classification (cars, motorcycles, trucks, etc.), enabling richer contextual understanding of incidents. This level of detail supports more informed decision-making in both emergency response and traffic management.
- We introduce a collaborative incident reporting mechanism where intelligent agents share contextual insights—including accident location, vehicle categories, and scene-level interpretation—to provide actionable information for real-time emergency response as well as post-incident analytics.
- The proposed framework is rigorously validated on real-world traffic video datasets. Experimental results demonstrate its effectiveness in achieving high detection accuracy, reliable multi-agent coordination, and practical applicability in smart traffic systems.

The remainder of this paper is organized as follows: Section 2 reviews related work on accident detection, multi-agent systems, and vision-language models. Section 3 describes the proposed framework, including the system architecture, data preprocessing, and model integration. Section 4 presents the evaluation of the experiments. Section 5 presents the experimental results, performance evaluation, confusion matrices, and visualizations. Finally, Section 6 concludes the study. Section 7 suggests directions for future research.

## 2. Related Work

Accident detection evolved from manual to smart systems using computer vision and machine learning. Methods include traditional techniques, computer vision-based classifiers, video LLMs, and MASs, each with unique strengths and limits. The proposed multi-layered framework combines these approaches for more collaborative and accurate detection. A comparative summary of these methods is presented in Table 1, which highlights their key features, advantages, and limitations.

### 2.1. Conventional Accident Detection

Traditional accident detection relies on human input and basic sensors, often delaying emergency responses due to manual alerts via hotlines or control centers. Internet of Things (IoT)-based accident detection systems, such as those by [7,8], often use sensors, GPS, and accelerometers but rely on human input or lack rescue coordination. To complement detection, advanced communication technologies [9] enable post-incident management by dynamically disseminating information and guiding vehicles to reduce congestion. While IoT-based systems [10] primarily focus on accident detection and immediate response, lane-level HD intersection mapping with low-cost roadside LiDAR extends these capabilities by supporting precise traffic accident evaluation and prevention. Similar to UAV traffic management [11], where AI-based methods enhance conflict detection and resolution, intelligent IoT and mapping technologies advance accident detection, response, and prevention in road networks. Ref. [12] also requires manual hospital alerts. In contrast, ref. [13] compares an SVM and PNN for real-time detection using traffic, weather, and loop data, applying SMOTE for imbalance; an SVM offers better accuracy, while a PNN detects incidents shortly after they occur. Ref. [14] enhances prediction by transforming multi-class classification into binary tasks using XGBoost, followed by a neural network, achieving strong results on real-world data. While IoT solutions are valuable, they are costly and less accurate—limitations that can be addressed using standalone AI or AI–IoT integration. Traditional ML models also struggle with imbalanced data and complex spatial-temporal patterns.

### 2.2. Modern Methods of Accident Detection

Modern accident detection uses IoT and deep learning (e.g., YOLO, Faster R-CNN, Swin Transformers) for real-time, accurate analysis from CCTV, GPS, and sensor data. Traditional methods like image subtraction [15] fail under lighting changes and lack temporal awareness [16,17].

#### 2.2.1. Computer Vision-Based Detection

Vision Transformers (ViTs) outperform traditional models in computer vision, offering scalability and efficient handling of sparse data [18,19], with the Swin Transformer excelling in dementia detection [20] and ViTs showing effectiveness in medical imaging, such as diabetic retinopathy detection [21,22]. YOLO-based models like LampRepSE-YOLO and YOLO-Vehicle-Pro lead real-time object detection for autonomous vehicles, achieving high accuracy through multimodal fusion [23,24], while integrating lightweight architectures like MobileNet improves speed and efficiency for real-time navigation [25]. In [26], an enhanced VGG19 model achieves 96% accuracy and a 0.99 AUC for accident classification. The Smart Fire Detection System (SFDS) using YOLOv8 in [3] achieves 97.1% precision, with applications in public safety and environmental monitoring. YOLO variants like YOLO-CCS [24] and DAN-YOLO [27] enhance performance for intelligent transport systems, while orthogonal attention methods improve occluded object detection in surveillance [28,29], demonstrating YOLO’s growing impact in real-time safety and mobility.

#### 2.2.2. MASs in Intelligent Traffic System

MASs consist of independent, adaptable agents working together to achieve shared or individual goals, enhancing traffic management by optimizing traffic flow and control, especially when integrated with the IoT for intelligent traffic light control [30]. Multi-Agent Reinforcement Learning (MARL) improves traffic signal optimization and routing for autonomous vehicles, outperforming traditional methods [4], while MASs can prioritize emergency vehicles for quicker response times [31]. However, CNN models, though strong in feature extraction, face challenges in dynamic traffic environments, such as overfitting and slow performance [32,33]. Recent advancements in lightweight feature extraction and attention mechanisms [34] enhance CNNs, and when integrated into MASs, these innovations improve real-time collaboration, boosting accuracy and efficiency in accident detection and surveillance.

#### 2.2.3. Classifiers

Deep learning classifiers like YOLO, ResNet, and MobileNet are crucial for real-time accident and object detection in traffic. YOLOv11 further improves detection with enhanced adaptability, an optimized architecture, and better feature extraction, supporting tasks like posture estimation and instance segmentation [5]. ResNet50 uses residual learning to train deep networks and overcome vanishing gradients [35], while a ViT leverages self-attention on image patches for strong global feature learning [36]. VGG16, with its 3 × 3 filters, is effective in image recognition despite its depth [36], and MobileNet offers efficient performance on embedded systems using depthwise separable convolutions [37].

In the proposed CIRS framework, YOLOv11 serves as the main classifier, enabling real-time vehicle and accident detection with high accuracy and speed, which are crucial for efficient traffic monitoring.

#### 2.2.4. Vision Language Model

Recent advancements in multimodal LLMs have enhanced video understanding and Visual Question Answering (VQA). Molmo, developed by AllenAI, offers strong performance in image-based tasks but lacks temporal modeling for video comprehension [38]. LLaVA-OneVision, a unified model for image and video understanding, achieves competitive video QA results (62% on MC-VQA), though its frame-based sampling limits temporal reasoning [39]. Qwen2-VL, from Alibaba, excels in document-centric VQA (96.5% on InfoVQA) and long video comprehension but is restricted in its 72B version [40].

The VideoLLaMA series, developed by DAMO Academy, advances video comprehension by integrating audio–visual features. VideoLLaMA2 uses a Spatial-Temporal Convolution connector, achieving 54.6% on MVBench [41], while VideoLLaMA3 adopts an image-centric training approach to improve video reasoning. However, long video comprehension remains a challenge, with GPT-4o excelling in multi-step temporal reasoning [6]. Open models like InternVL2.5 and Qwen2-VL are closing the gap with proprietary systems such as GPT-4V and Claude 3.5, though challenges in long-sequence processing and temporal reasoning persist.

#### 2.2.5. Challenges in Current Traffic Incident Classification Approaches

Accident detection systems often face delays, high false positives, and limited scalability, especially in crowded or complex environments. They typically lack coordination among traffic entities and overlook contextual factors like weather or road conditions, reducing accuracy and delaying responses. To overcome these issues, the proposed CIRS framework integrates YOLOv11 for real-time detection, VideoLLaMA3 for semantic video understanding, and a multi-agent system for coordinated, decentralized response—enhancing accuracy, context awareness, and responsiveness.

**Table 1:** Comparison of Detection and Traffic Management Approaches.

Category	Reference	Method	Technology	Key Features	Limitations	Performance Metrics
Traditional	–	Manual Reporting	Human and Hotline	Low-cost alerts via hotline	Slow response time	Time and Accuracy
	[7]	Sensor-Based	Vibration Sensor, AT89S52	Auto-alert via vibration	No emergency handling	Accuracy and False Alarms
	[42]	IoT-Based	GPS, Accelerometer, GSM	Detects severity and location	Costly, requires network	Accuracy and Latency
	[43]	ML-Based	XGBoost and ANN	Predictive accident classification	Limited to known patterns	F1-score
Modern	[3]	Fire Detection	YOLOv8 and SFDS	Detects fire using deep learning	High computational cost	Precision, Recall, and F1-score
	[26]	Image Classification	VGG19 (TL)	DL-based accident analysis	Needs labeled data	Accuracy, Precision, Recall, and F1-score
MAS	[4]	Multi-Agent RL	RL agents	Smart routing with RL agents	High complexity	Efficiency, Waiting Time
	[30]	Adaptive Signals	IoT + MAS	Real-time traffic signal adaptation	Needs IoT setup	Flow, Adaptivity
Classifiers	[5]	YOLOv11	Enhanced YOLO	Real-time detection	Hardware optimization needed	Accuracy, Speed
	[44]	ResNet50	Deep CNN	Handles vanishing gradients	High computational cost	Accuracy, Efficiency
	[22]	ViT	Vision Transformer	Global feature learning	Requires large dataset	Accuracy, Efficiency
	[36]	VGG16	CNN Model	Deep CNN, simple architecture	High demand	Accuracy, Complexity
	[37]	MobileNet	Lightweight CNN	Mobile-optimized detection	Lower accuracy	Latency, Power
VLMs	[38]	Molmo	PixMo and 2D Pointing	VLM-based video QA	Weak temporal modeling	VQA Accuracy and Speed
	[39]	LLaVA-OneVision	SigLIP and Qwen2	Multimodal video-text analysis	Struggles with fine-grained tasks	VQA Score
	[40]	Qwen2-VL	RoPE Transformer	Strong document-based VQA	Needs real-world testing	VQA Score

## 3. Proposed Methodology

The multi-agent-based CIRS proposed framework specifically for video-based accident detection is meticulously designed to detect and classify traffic accidents in real-time situations. This CIRS incorporates multi-agent technologies such as leveraging the YOLOv11 model for visual accident detection and classification, employing LLaVA-3 for video-based reasoning, and decision-making for intelligent emergency response management, thus establishing a comprehensive and sophisticated framework for accident detection and emergency response.

### 3.1. CIRS Framework Architecture

**Figure 1:**
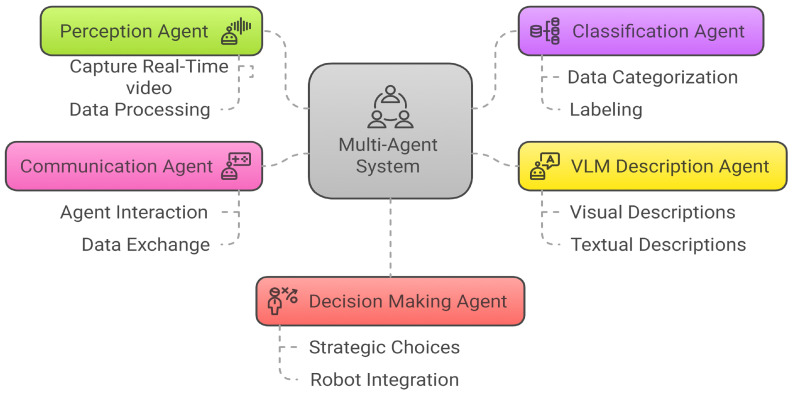
Multi-Agent CIRS framework, wherein different agents are responsible for specific tasks, ensuring a structured and flexible workflow.

The CIRS framework comprises multiple intelligent agents that collaboratively analyze video streams to detect accidents and support response management. Each agent operates autonomously within its specialized role while exchanging information to enhance the overall precision and responsiveness of the system collectively. In the proposed CIRS framework, discrete agents are tasked with specific responsibilities, thus ensuring an organized workflow as shown in Figure 1.

#### 3.1.1. Perception Agents (Camera Nodes)

Each surveillance camera functions as an independent entity, persistently acquiring and analyzing video frames to identify potential features.

#### 3.1.2. Classification Agent

Employ deep learning models, such as YOLOv11, to execute binary classification on each individual frame, thus determining the occurrence of accidents.

#### 3.1.3. Description Agent

Once an accident is detected, the description agent generates captions and identifies the types of vehicles involved, such as cars, bikes, and trucks. This process contributes to a more comprehensive analysis of the accident and facilitates the assessment of the severity level by utilizing the VLM.

#### 3.1.4. Communication Agent

Coordinate information exchange between other agents and emergency response units for timely intervention.

#### 3.1.5. Decision Agent (Emergency Response Coordination)

After processing the accident information, decision agents assess the severity and initiate appropriate response actions, such as alerting emergency services.

### 3.2. CIRS Processing Pipeline

**Figure 2:**
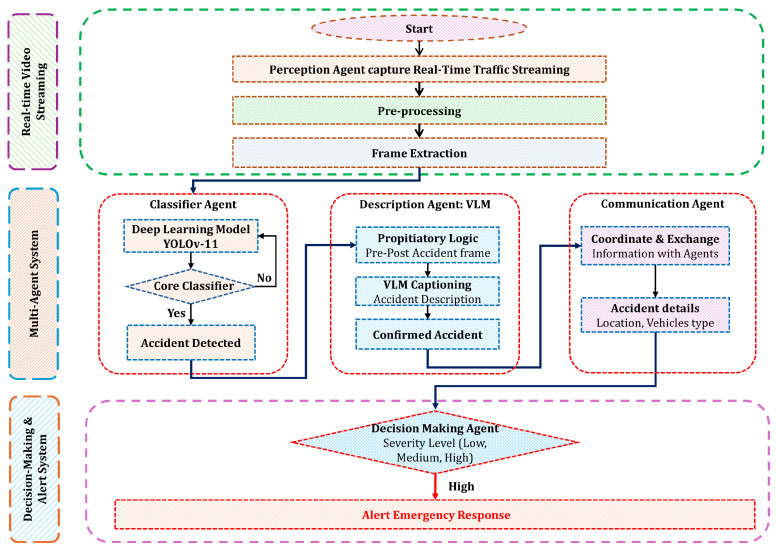
The Multi-Agent CIRS framework enables real-time, decentralized accident detection through video-based intelligence. By facilitating efficient collaboration among intelligent agents, it ensures rapid and accurate detection, classification, and response, enhancing overall road safety.

The CIRS framework follows a systematic processing pipeline to ensure seamless accident detection and response management. The approach is illustrated in Figure 2.

#### 3.2.1. Perception Agent–Input Stream Acquisition and Frame Pre-Processing

The perception agent is responsible for acquiring the input video stream and pre-processing frames for further analysis. This process begins by interfacing with real-time traffic surveillance cameras, in order to ensure high-quality feed acquisition with minimal latency. We utilize fixed, predefined camera deployment locations—such as road intersections—to enable agents to infer the scene location contextually, which reflects real-world infrastructure where cameras are statically installed. Let the video stream be represented as V, where *t* is the time. Equation (1), however, shows the extracted frames from the video stream as $F^{\prime }$ at a certain time instant.

(1) $$V=\{F^{1},F^{2},F^{3},F^{4},\ldots ,F^{t}\}$$

(2) $$F^{\prime }=Q\left(V(t)\right),\;\forall t\in \left\{1,2,\ldots ,t\right\}$$

where Q(·) is the pre-processing function that standardizes frames in Equation (2). After acquisition, frames are extracted optimally to balance computational efficiency and real-time detection accuracy. A rolling buffer mechanism stores a configurable number of frames preceding an event, providing pre-event context for accident analysis. The buffer dynamically maintains a sliding window of frames, ensuring that critical moments before an incident are preserved. These pre-processed frames are then forwarded to the accident classifier agent for classification.

#### 3.2.2. Accident Classification Agent: Core Classifier

**Figure 3:**
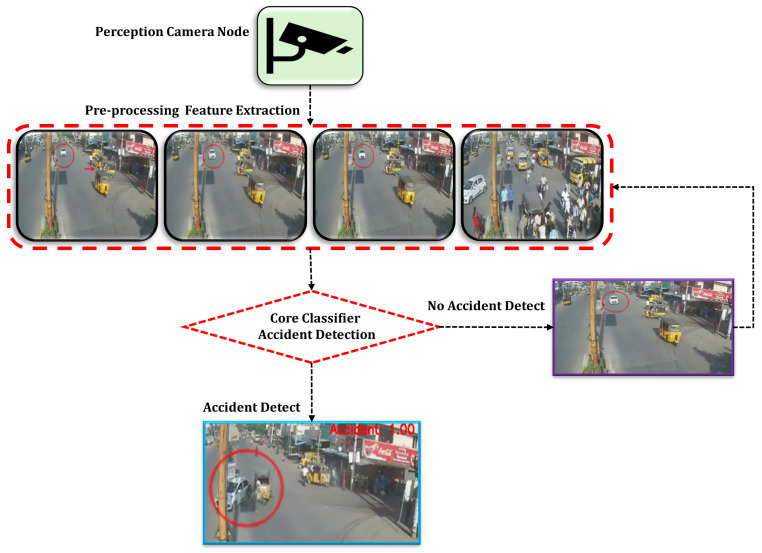
The accident detection agents are responsible for classifying each frame as either ‘Accident’ or ‘No Accident,’ thereby ensuring accurate identification of traffic incidents in real time.The red circle represents the accident in the frame.

The classifier agent utilizes the YOLOv11 model to categorize video frames into two primary classes, Accident and No Accident, as visualized in Figure 3, which is based on footage from the youtube video. YOLOv11, an open-source object detection model, was used for classifying accident and non-accident scenarios. While the architecture was not modified, it was trained on our custom accident dataset for improved contextual performance.

The classifier agent analyzes each frame to assess that an accident has occurred, and is mathematically presented in Equation (4).

(3) $$\;A_{t}=C\left(F_{t}\right),\;\forall t\in \left\{1,2,\ldots ,t\right\},$$

(4) $$A_{t}=\left\{\begin{array}{cc} 1, & \mathrm{if}\;\mathrm{an}\;\mathrm{accident}\;\mathrm{is}\;\mathrm{detected}\\ 0, & \mathrm{otherwise} \end{array}\right.$$

The classification function C(·) is represented as the YOLOv11 model in Equation (3), and $A_{t}$ represents the classification output for each frame $F_{t}$. To optimize the efficiency of event classification, a dynamic frame buffering strategy comprises the following:

A *core classifier* (such as the You Only Look Once model) to detect potential accidents in the frames.

*Dynamic Frame Buffering* maintains a rolling buffer of frames for 1s (adjustable) before accident detection. It ensures that the pre-event context is saved for comprehensive analysis.

*Confidence-Based Triggering* initiates video saving when the confidence of an “Accident” classification exceeds a configurable threshold (e.g., 55%).

*Proprietary Logic for Redundancy Avoidance* introduces a cooldown period of 5 s between consecutive saves to prevent redundant video clips.

(5) $$\;V_{accident}=V\left(t\right),\;\forall t\in \left[t_{start},t_{end}\right],$$

When an accident is detected, a proprietary logic module governs pre- and post-event video saving, ensuring that relevant frames, such as mathematical formulations given in Equation (5), are retained while discarding redundant ones.

The proposed proprietary logic ensures the efficient and accurate saving of video clips around the detected accident events. It dynamically adapts to the detection confidence, minimizes redundant saves, and provides a seamless mechanism for capturing pre- and post-event frames.

#### 3.2.3. Accident Description Agent: VLM

The Description Agent, powered by a VLM, is responsible for generating textual descriptions (captions) T of detected accident videos $V_{\mathrm{accident}}$ by analyzing visual and contextual features $C_{\mathrm{context}}$ extracted from CCTV footage as a formulation given in Equation (6). It serves as a bridge between machine understanding and human-readable reporting.

(6) $$T={\mathrm{VLM}}_{\mathrm{Decoder}}\left(f\left(V_{\mathrm{accident}}\right)⊕g\left(C_{\mathrm{context}}\right)\right)$$

whereas *f* is the visual encoder, *g* is the contextual encoder, and ⊕ refers to the concatenation process used to combine visual and contextual features. The accident description is generated using the ${\mathrm{VLM}}_{\mathrm{Decoder}}$ vision-language model represented in Equation (6).

#### 3.2.4. Communication Agent

The Communication Agent is crucial in managing the flow of information through a multi-agent system during accident detection and response. The process starts with data coordination and interchange among agents, which is mathematically modeled in Equation (7) using the coordination function $h_{\mathrm{coord}}$. The function computes the exchanged information $I_{ij}\left(t\right)$ by combining the states or messages of two interacting agents, $S_{i}\left(t\right)$ and $S_{j}\left(t\right)$. In Equation (8), $I_{\mathrm{total}}\left(t\right)$ represents the total information transferred inside the multi-agent system at a given moment *t*, which is the sum of all individual conversations between each pair of agents.

(7) $$I_{ij}\left(t\right)=h_{\mathrm{coord}}\left(S_{i}\left(t\right),S_{j}\left(t\right)\right)$$

(8) $$I_{\mathrm{total}}\left(t\right)=\sum_{i=1}^{n}\sum_{j=1}^{n}I_{ij}\left(t\right)$$

Thus, after coordinate information, the agent processes *accident-specific details*, such as the *location* and *type of vehicles* involved.

Furthermore, as shown mathematically in Equation (11), $V_{\mathrm{type}}$ denotes a collection of involved vehicle types, such as cars, buses, or trucks, while $L_{\mathrm{acc}}$ in Equation (10) relates to the geographic coordinates of the accident site. These details are collectively represented as in Equation (9).

(9) $$D_{\mathrm{acc}}=\left(L_{\mathrm{acc}},V_{\mathrm{type}}\right)$$

(10) $$L_{\mathrm{acc}}=\left(x,y\right)$$

(11) $$V_{\mathrm{type}}=\left\{v_{car},v_{bus},\ldots ,v_{truck}\right\}$$

Equation (12), provides a communication function $C_{\mathrm{agent}}\left(t\right)$ that processes all of the coordination-processed data. This function captures both the inter-agent communication and the distribution of accident-specific information for subsequent action. As demonstrated quantitatively in Equation (13), which calculates the delay between sending and receiving a message, the *latency* has a positive impact on the communication process.

(12) $$C_{\mathrm{agent}}\left(t\right)=g\left(I_{ij}\left(t\right),D_{\mathrm{acc}}\left(t\right)\right)$$

(13) $$\Delta T_{\mathrm{comm}}=T_{\mathrm{recv}}-T_{\mathrm{send}}$$

#### 3.2.5. Decision-Making Agent

The Decision-Making Agent (DMA) is activated whenever the communication agent has provided validated accident information, such as location, involved vehicle types, and the number of vehicles involved in the accident. The DMA assesses the severity of the occurrence (Equation (14)) and figures out an acceptable emergency response strategy.

(14) $$S_{\mathrm{level}}=h\left(D_{\mathrm{acc}},V_{\mathrm{type}},N_{\mathrm{vehicles}}\right)$$

Accident detail represent the $D_{\mathrm{acc}}$, $V_{\mathrm{type}}$ represents the set of vehicle types, and $N_{\mathrm{vehicles}}$ is the number of vehicles involved. The function h(·) maps these features to categorical severity levels {Low,Moderate,Critical}.

##### Emergency Response Decision

The Emergency Response Decision process is a crucial function of the Decision-Making Agent, which activates once subsequent agents have identified and confirmed an accident. The agent performs a decision-making function defined in Equation (15) while acquiring information such as the accident location $L_{\mathrm{acc}}$, the accident severity level $S_{\mathrm{level}}$, and the locations and current availability of emergency response units $R_{\mathrm{units}}$.

(15) $$E_{\mathrm{response}}=f_{\mathrm{decision}}\left(S_{\mathrm{level}},L_{\mathrm{acc}},R_{\mathrm{units}}\right)$$

In order to select the most appropriate and timely response strategy, assess the accident’s contextual factors. The agent assigns the most effective unit based on location and projected response time if the severity level indicates a moderate or critical condition. The decision mechanism makes sure that high-severity events are handled with the proper urgency, in addition to decreased response latency. The outcome of the process is a structured emergency dispatch plan that directs specific response units to the accident location with clear instructions, thereby enhancing the overall effectiveness of the intelligent traffic management system.

## 4. Experimental Evaluation

This section presents the experimental results used to evaluate the effectiveness of the proposed CIRS model. The evaluation was conducted through three main stages: (a) data preprocessing of the traffic dataset, (b) accident classification to identify accident-related frames, and (c) the generation of descriptive narratives using a vision-language model. In particular, we detail the real-world traffic dataset employed in the experiments, as well as the evaluation metrics commonly adopted for accident detection tasks.

### 4.1. Dataset

This research study utilized the CCTV dataset that comprises 157 videos from public and proprietary archives. To ensure consistency in viewpoint and motion, dashcam and handheld footage were deliberately excluded. CIRS was tested under varied angles, lighting, and moderate weather, showing good robustness. Extreme conditions (e.g., fog, low light) remain to be addressed on future work.

The dataset, which is 4.28 GB in size, is designed to support the development of intelligent transportation systems (ITSs) by offering diverse and representative traffic scenarios. The Central Police Office in Karachi, Sindh, provided a sizable amount of the dataset, which included videos of various accident cases and traffic congestion. Additional CCTV videos from public and institutional sources present a variety of urban traffic conditions. All experiments were conducted using accident-only video scenarios to evaluate model performance in critical conditions. Moreover, accident scenarios were broadly defined to include both major collisions (e.g., multi-vehicle crashes) and minor incidents such as bike falls, as long as they disrupt normal traffic flow. This definition was consistently applied during dataset annotation to ensure that the model learns to recognize diverse real-world accidents. A detailed summary of the dataset characteristics is provided in Table 2.

#### 4.1.1. Data Preprocessing and Annotation

Videos were sampled at a rate of 0.5 frames per second (FPS), resulting in the extraction of one frame every two seconds. This rate was deliberately selected to ensure temporal diversity while avoiding excessive redundancy, particularly in long-duration surveillance footage where consecutive frames often show minimal variation. The selected sampling frequency offers a balance between representativeness and efficiency by capturing key temporal transitions across the video sequence without overwhelming the dataset with highly similar frames. While a higher sampling rate could potentially capture finer-grained accident dynamics, the current rate was found to be sufficient for the scope of scene-level accident understanding and captioning in this study. Each frame was annotated using the Computer Vision Annotation Tool (CVAT) and assigned labels as either Accident or No Accident. According to CVAT analytics, the annotation process required approximately 215 h. Table 3 shows the dataset subsequently divided into training (80%) and validation (20%) subsets and formatted to be compatible with the YOLOv11 framework.

#### 4.1.2. Dataset Statistics

**Figure 4:**
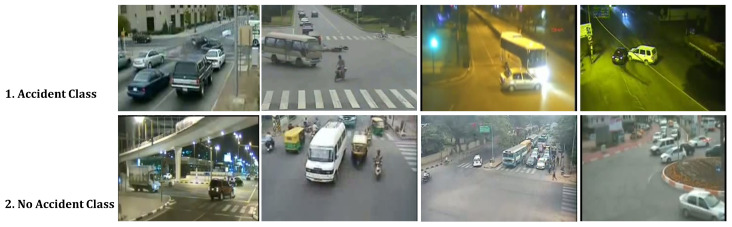
Visual representation of the dataset distribution, illustrating the classification of frames into “Accident” and “No Accident” categories for supervised model training.

The final preprocessed dataset comprises two classes, as illustrated in Figure 4, with approximately 5200 frames labeled as Accident and 4800 frames labeled as No Accident. This distribution yields a nearly balanced binary classification setup, making it suitable for training a robust supervised model.

### 4.2. Implementation Setup and Environment

To setup the hardware configuration required as shown in Table 4, the experiments were conducted on a high-performance workstation configured with an an Intel ^®^ Core™ i9-13900 (13th Gen) processor (manufactured in Ho Chi Minh City, Vietnam) and 64 GB of DDR RAM. The system was equipped with a Zotac^®^ (manufactured in Hong Kong, China), NVIDIA GeForce RTX 4090 Trinity GPU (Santa Clara, CA, USA), featuring 24 GB of GDDR6X VRAM. Additionally, a 1 TB NVMe PCIe Gen4 Solid State Drive (SSD) was used for high-speed data access. All experiments for the proposed CIRS framework were performed using Python 3.8 with CUDA version 13.12 and the Pytorch 2.3.1 framework.

### 4.3. Training and Validation of the YOLOv11-Based Model

The classification agent was trained on a pre-trained YOLOv11 architecture, selected for its robust feature extraction capabilities. The training configuration parameter is detailed in Table 5. The classifier agent was trained for 500 epochs, with the batch size dynamically adjusted between 32 and 64 depending on GPU memory availability. Optimization was performed using Stochastic Gradient Descent (SGD), employing a learning rate schedule that incorporated both warm-up and cosine decay to promote stable convergence. The Ultralytics YOLO library handled dataset processing and hyperparameter tuning, with an initial learning rate set of $3\times 10^{-3}$. All training was executed on CUDA-enabled GPUs to ensure computational efficiency and high classification accuracy.

### 4.4. Evaluation Metrics

The performance of the proposed CIRS model is evaluated using classification, computational, and vision-language model (VLM) metrics. Classification performance was assessed using standard metrics such as precision, recall, F1-score, and accuracy [45] to measure the effectiveness of accident detection model. However, computational efficiency is evaluated through inference time, model size, and GPU utilization. By comparing the quality of descriptive text generated by the VLM, vision-language model, to the generated outputs against human-written references, natural language generation metrics such as ROUGE [46], BLEU [47], and METEOR [48] serve as key performance indicators of the descriptive text produced by the vision-language model. Table 6, provides a brief summary of the formulas used for the evaluation metrics.

## 5. Results and Discussion

We conduct a comprehensive analysis of the experimental results for the proposed CIRS framework. The evaluation encompasses multiple components, including the benchmarking of the classification model, the performance assessment of the selected YOLOv11-based classifier agent, and overall framework efficiency, with a focus on reasoning capabilities provided by the vision-language model (VLM) agent.

### 5.1. Comparative Performance Analysis of Classification Models

**Figure 5:**
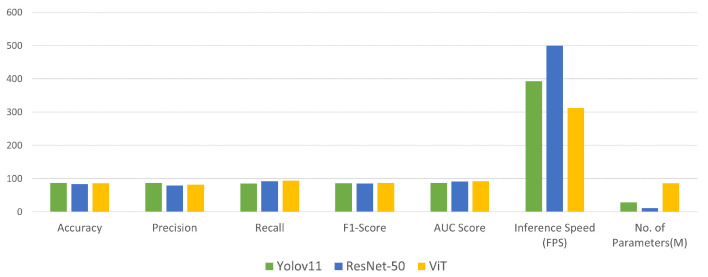
Bar plot illustrating key performance metrics of the YOLOv11 model, including the number of parameters, accuracy, precision, recall, F1-score, AUC score, and inference speed. This visualization provides a comparative overview of the model’s classification effectiveness, computational efficiency, and real-time applicability.

We evaluated YOLOv11, ResNet-50, and a Vision Transformer (ViT) for accident detection, comparing classification accuracy and computational efficiency. As illustrated in Figure 5, and detailed in Table 7, the models were assessed using accuracy, precision, recall, F1-score, inference speed, and model complexity.

YOLOv11 emerged as the most balanced performer, achieving the highest precision (86.6%), along with a strong accuracy and F1-score. Its fast inference speed (393 FPS) and moderate model size (28.34 M parameters) make it well-suited for real-time, resource-constrained accident detection scenarios. The ViT achieves the highest recall (93.77%), indicating strong detection capability, but its slower inference speed (312.5 FPS) and large model size (85.80 M parameters) hinder real-time deployment. Its lower precision (81.65%) also increases the risk of false positives. ResNet-50, while it is the fastest (500 FPS) and most lightweight (11.18 M parameters), suffers from lower precision (78.97%), reducing its reliability. Overall, YOLOv11 offers the best balance of accuracy, speed, and model size, making it the most suitable choice for real-time accident detection in intelligent traffic monitoring systems.

### 5.2. Yolov11 Classification Agent Performance Results

**Figure 6:**
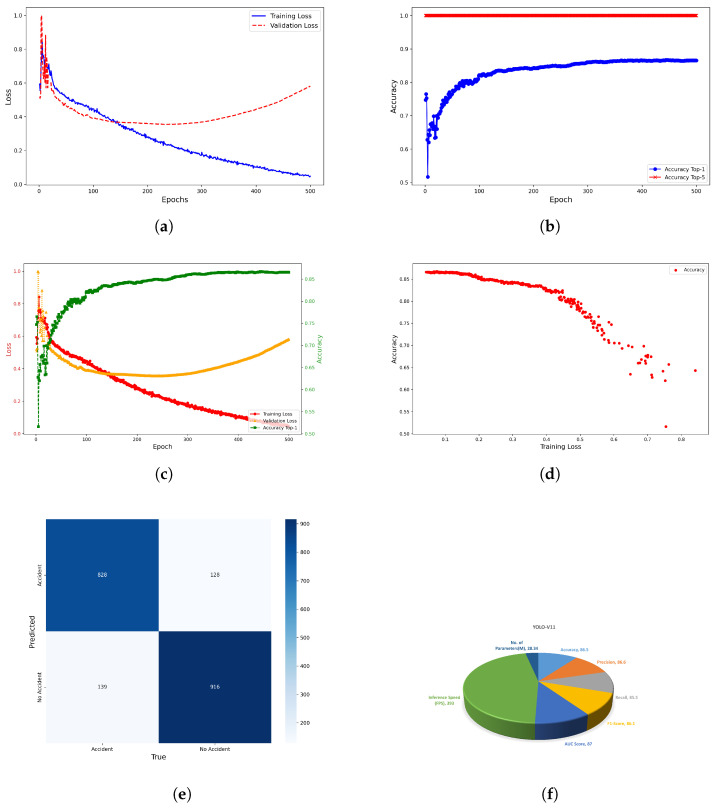
Classifier agent performance evaluation of YOLOv11 across different metrics: loss, accuracy, convergence behavior, classification outcomes, and overall efficiency metrics. (**a**) Training and validation loss curves showing YOLOv11 model convergence over epochs. (**b**) Top-1 and top-5 accuracy trends over 500 epochs for binary accident detection. (**c**) Epoch-wise training/validation loss and accuracy curves highlighting learning progression. (**d**) Scatter plot of training loss vs accuracy indicating model generalization. (**e**) Confusion matrix presenting YOLOv11’s classification performance on the validation dataset. The matrix shows the number of true positives (TPs), true negatives (TNs), false positives (FPs), and false negatives (FNs), reflecting the model’s ability to distinguish between accident and non-accident frames. (**f**) Pie chart summarizing key performance indicators such as the number of parameters, accuracy, precision, recall, F1-score, AUC score, and inference speed, offering a comprehensive visual representation of the model’s efficiency and effectiveness in real-time accident detection.

Following a comparative evaluation with ResNet-50 and a Vision Transformer (ViT), YOLOv11 was selected due to its optimal balance of precision, inference speed, and computational efficiency. This section provides an in-depth analysis of YOLOv11’s performance in the binary classification task of detecting accident versus non-accident frames as shown in Figure 6.

#### 5.2.1. Training Performance and Model Convergence

We trained YOLOv11 with consistent learning over 500 epochs. As detailed in Table 8, both training and validation losses steadily declined, highlighting the model’s ability to generalize effectively. It achieved an impressive top-1 accuracy of 86.5% and a perfect top-5 accuracy of 100%, confidently classifying the correct class in every prediction even within the constraints of a binary classification task. These findings demonstrate YOLOv11’s robustness and reliability for real-time accident detection. The learning rate was adaptively adjusted from $1.594\times 10^{-4}$ to $1.198\times 10^{-4}$ during training, enabling fine-tuning as the model approached optimal performance. These logs reflect the stability and convergence of YOLOv11, which is essential for dependable real-time accident detection. Such consistency establishes a strong foundation for subsequent evaluation under real-world conditions.

#### 5.2.2. Training Dynamics and Validation Loss Analysis of YOLOv11

Figure 6a illustrates the training and validation loss curve of YOLOv11 over 500 epochs. The training loss (blue) demonstrates a steadily downward trend, indicating effective learning. The validation loss (red) initially decreases but begins to rise after approximately 150 epochs, suggesting the onset of mild overfitting. Nevertheless, core performance metrics such as accuracy, precision, and recall remained stable throughout training, implying that the overfitting had minimal impact on the model’s generalization. Instances of NaN values observed in the validation loss logs are considered outliers and do not influence the overall assessment. These observations highlight the importance of jointly monitoring loss curves and evaluation metrics, particularly in safety-critical applications like accident detection.

#### 5.2.3. Accuracy Trends and Model Convergence Analysis

Figure 6b presents the top-1 and top-5 accuracy trends of YOLOv11 over 500 training epochs. The top-1 accuracy begins at 74.7%, exhibits initial fluctuations due to model adaptation, and stabilizes between 86% and 87% from epoch 444 onward. These early variations are characteristic of the learning phase as the model refines its parameters. The top-5 accuracy consistently remains at 100%, which is expected in binary classification tasks where the correct label is always among the top predictions. This stability highlights YOLOv11’s robust capability in capturing discriminative features that are essential for accurate accident detection. Figure 6c shows that while training loss decreases steadily, validation loss starts rising after epoch 250, indicating potential overfitting. However, top-1 accuracy continues to improve, stabilizing around 86–87%, demonstrating effective learning and consistent performance.

#### 5.2.4. Correlation Between Training Loss and Accuracy

Figure 6d presents a scatter plot illustrating the relationship between training loss and accuracy over training epochs. The line-type red dot represents an individual epoch, revealing a distinct inverse correlation—accuracy tends to increase as training loss decreases. Notably, the loss drops below 0.2, and the accuracy stabilizes in the range of approximately 86–87%, suggesting that the model has achieved stable generalization performance. This trend indicates effective learning dynamics and reflects a well-optimized training process for the YOLOv11 model in the context of accident detection.

#### 5.2.5. Confusion Matrix Analysis

Figure 6e and Table 9 provide a comprehensive overview of the classification performance of the YOLOv11 model on the validation dataset. The confusion matrix highlights the model’s ability to accurately differentiate between accident and non-accident frames. Specifically, YOLOv11 correctly classified 828 accident frames as true positives (TPs) and 916 non-accident frames as true negatives (TNs). However, the model failed to detect 139 accident cases (false negatives, FNs) and incorrectly identified 128 non-accident frames as accidents (false positives, FPs). These results demonstrate that while the model exhibits a strong classification capability, some misclassifications remain, indicating potential areas for further refinement.

The model missed approximately 14% of actual accidents (139 out of 967), indicating a need for threshold adjustment to improve recall. Additionally, 12% of non-accident frames (128 out of 1044) were falsely classified as accidents. Despite the fact that this is not optimal, this level of false positives may be tolerable in real-time applications where prioritizing the detection of true accidents is critical. The confusion matrix highlights the trade-offs between false positives and false negatives in safety-critical tasks like accident detection.

#### 5.2.6. Evaluation Metrics for Accident Detection

YOLOv11’s performance on accident detection was evaluated using key classification metrics: precision, recall, and F1-score for the Accident class. The model achieved a precision of 0.866, indicating that 86.6% of predicted accident instances were correct, thus reflecting a low false-positive rate. The recall was 0.856, meaning that 14.4% of actual accidents were missed—a critical consideration in safety-sensitive applications. The F1-score of 0.861 demonstrates a strong balance between precision and recall, highlighting the model’s robustness in managing the trade-off between missed detections and false alarms. These results underscore the effectiveness and reliability of YOLOv11 in real-world accident detection scenarios. Figure 6f presents a summary pie chart illustrating the key performance indicators, providing a visual overview of the model’s classification capabilities.

### 5.3. Vision to Insight VLM-Based Accident Description and Assessment

As we analyze, YOLOv11 demonstrates high performance in detecting accidents through visual input; however, it lacks the capability to provide contextual information such as the severity of the incident, the number of vehicles damaged, or surrounding environmental conditions. To address this limitation, the integration of a vision-language model enables the generation of detailed natural language descriptions, offering deeper insights into each event. This fusion enhances the ability to prioritize incidents, differentiate levels of crash severity, and recognize critical features such as overturned vehicles or hazardous surroundings. Furthermore, the system facilitates post-accident processes by automating the generation of incident reports, significantly reducing manual workload. The combination of YOLOv11 and an VLM ultimately improves situational awareness, accelerates decision-making, and increases overall response efficiency in accident management systems.

**Figure 7:**
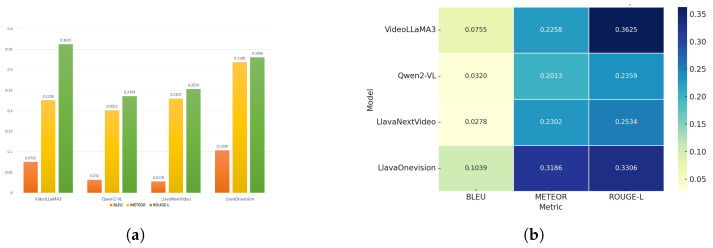
Visual comparison of vision-language model (VLM) performance. (**a**) A bar chart summarizing BLEU, METEOR, and ROUGE-L scores, while (**b**) displays a heatmap of evaluation metrics. (**a**) Quantitative comparison of various vision-language models (VLMs) based on standard natural language generation metrics: BLEU, METEOR, and ROUGE-L. (**b**) Heatmap showing evaluation metrics.

Moreover, for accurate accident detection, we evaluated four video-capable language models, VideoLLaMA3, Qwen2-VL, LlavaNextVideo, and LlavaOneVision, using standard metrics (BLEU, METEOR, ROUGE-L), summarized in Table 10 and Figure 7, which provides a visual comparison of performance across different models. In Figure 7a, LlavaOneVision demonstrated the highest evaluation scores (BLEU: 0.1039, METEOR: 0.3186, ROUGE-L: 0.3306), indicating strong generative capabilities in describing accident scenes. Despite these metrics, the model exhibited a tendency to hallucinate non-existent scene elements such as fabricated vehicles or license plates, which undermines its reliability for deployment in real-world accident reporting scenarios. In contrast, VideoLLaMA3 offered a better balance between descriptive quality and factual accuracy, with competitive scores (BLEU: 0.0755, METEOR: 0.2258, ROUGE-L: 0.3625) and fewer hallucinations. Qwen2-VL and LlavaNextVideo showed moderate results, but occasional misinterpretations limited their suitability for high-stakes accident analysis.

The heatmap presented in Figure 7b further substantiates the robustness of VideoLLaMA3 across all evaluated metrics, reinforcing its suitability as the most practical candidate for real-world deployment. The experimental findings revealed that LlavaOneVision achieved the highest scores on standard language generation metrics, but it frequently introduced non-existent details such as fabricated accidents or license plates, thereby reducing its reliability for real-world applications. In contrast, VideoLLaMA3 demonstrated a more balanced performance, offering greater factual accuracy, clarity, and consistency in its descriptions. Based on strength, the VideoLLaMA3 agent was selected for integration with the YOLO-based accident detection system.

#### 5.3.1. Qualitative Evaluation of VideoLLaMA3 Captioning and Visual Understanding

**Figure 8:**
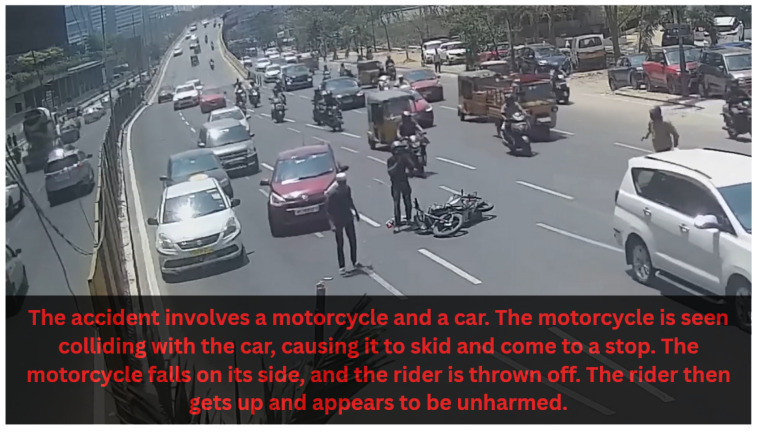
Qualitative results of VideoLLaMA3 demonstrate its capabilities in video frame captioning and visual scene understanding.

Figure 8 presents a qualitative evaluation of VideoLLaMA3, showcasing its effectiveness in generating accurate captions and interpreting visual content from video frames. The description agent performance is assessed by the ability to recognize objects, actions, and contextual relationships within dynamic scenes. We analyze the qualitative results, which indicate that VideoLLaMA3 is capable of producing contextually relevant and semantically rich descriptions of complex visual inputs.

#### 5.3.2. Overall CIRS Framework Performance for Emergency Responses

Table 11 presents the assessment of the overall performance of our CIRS framework. YOLOv11 detects accidents rapidly at 3–7 ms per frame, while VideoLLaMA3 takes 2–4 s per video for outputs. CIRS achieves an end-to-end response initiation time of approximately 3–5 s, which is significantly faster than traditional methods. The communication agent sends verified data like location and vehicle details to emergency teams, activating the DMA to evaluate incident severity and generate a dispatch plan. This performance highlights the potential of CIRS to drastically reduce emergency response time, thereby enhancing the chances of timely medical assistance and improving overall road safety outcomes.

### 5.4. Discussion

The proposed CIRS model represents a significant advancement in intelligent accident detection and response. By integrating a high-performance object detection model (YOLOv11) with a vision-language model (VideoLLaMA3), the system offers not only accurate accident identification but also rich, contextual understanding of each incident. This hybrid architecture addresses two major challenges in intelligent transportation systems: detection accuracy and the interpretability of incident data. Our evaluation shows that YOLOv11 achieves a top-1 accuracy of 86.5% in detecting accident frames. While this performance is strong, false negatives—instances where real accidents are missed—pose a greater risk than false positives, as they can delay emergency response. Although adjusting detection thresholds could improve recall, it may also increase false alarms, which would reduce system reliability. These results suggest more diverse training datasets that include a range of weather conditions, lighting environments, and accident scenarios. Future efforts may benefit from data augmentation, focal loss optimization, and active learning strategies to reduce missed detections and enhance model robustness.

The training process for YOLOv11 utilized a cosine annealing learning rate schedule, decaying from 0.0033 to 0.00012 over 500 epochs. The learning curve was generally stable, though a few validation runs showed NaN values, possibly due to noisy or anomalous inputs. On the captioning side, VideoLLaMA3 demonstrated a strong balance between language quality and factual accuracy, with BLEU, METEOR, and ROUGE-L scores of 0.0755, 0.2258, and 0.3625, respectively. In qualitative assessments, the model showed a consistent ability to identify objects, actions, and contextual relationships within dynamic scenes. In contrast, LlavaOneVision, scoring higher on automatic metrics (BLEU: 0.1039, METEOR: 0.3186, ROUGE-L: 0.3306), often introduced hallucinated details such as fabricated vehicles or license plates. These inaccuracies reduce its reliability for real-world deployment, especially in safety-critical applications like traffic monitoring.

By coupling a fast and accurate classifier with a semantically rich captioning model, CIRS achieves a scalable and interpretable solution for accident analysis. YOLOv11 enables immediate detection with minimal delay; however, VideoLLaMA3 adds contextual layers that are critical for understanding the nature, severity, and contributing factors of each incident. This integration enables advanced features such as automated alert generation, real-time dashboards, and post-incident analysis, all of which contribute to better decision-making for traffic authorities and emergency responders. In advance, the CIRS framework opens multiple directions for future research and development. Enhancing detection recall without compromising precision remains a priority, as is improving the factual grounding of generated descriptions, and expanding the proposed model capability to interpret more complex scenarios such as multi-vehicle collisions or hazardous weather conditions. Incorporating feedback loops from human experts and emergency services into the system could further refine accuracy through active learning.

In summary, the integration of YOLOv11 and VideoLLaMA3 within the proposed CIRS framework provides an effective approach to bridging the gap between raw visual detection and high-level semantic understanding. This study highlights the potential of vision-language models in critical real-world applications, setting the stage for more intelligent, interpretable, and responsive transportation systems.

## 6. Conclusions

This research study introduced a comprehensive multi-stage CIRS framework for automated accident detection and classification, integrating computer vision and natural language processing to enhance situational awareness and response efficiency. By combining with the lightweight, high-speed, and accurate detection capability-based YOLOv11 classifier, trained over 500 epochs, it achieved an accuracy of approximately 86.5%, demonstrating strong precision and recall and validating the effectiveness of the core detection stage. Each component in the framework from perception agents to the decision agent plays a distinct role in ensuring end-to-end functionality.

To further enrich system output, the vision-language model (VideoLLaMA3-7B) analyzes the saved video clips, generating detailed natural language descriptions and severity assessments. The final annotated accident frames provide an interpretable, actionable summary of each incident. Together, these components deliver an end-to-end solution that not only detects accidents with high accuracy but also offers qualitative insights into accident characteristics, enhancing the potential for faster and more informed emergency responses. The proposed CIRS architecture ensures scalability, adaptability, and operational reliability in diverse traffic environments. The integration of vision-language model within real-time detection demonstrates strong potential for improving automated accident detection and decision-making in intelligent transportation systems.

## 7. Future Work

We plan to enhance YOLOv11 and VideoLLM3 in our accident detection system by boosting recall while maintaining precision through advanced data augmentation, threshold adjustments, and exploring cost-sensitive loss functions like focal loss to reduce false negatives. For captioning, we aim to improve the VLM module’s expressive power by refining prompts and expanding token limits for richer accident descriptions, providing deeper insights. We will also enhance spatial interpretability using object detection or segmentation to display accident locations with bounding boxes in videos.

In future work, we will also introduce predictive capabilities that enable earlier intervention and proactive responses. Although the proposed CIRS framework effectively detects and describes accidents post-occurrence. One promising direction is the integration of Reinforcement Learning (RL) to develop predictive agents that are capable of identifying high-risk scenarios before an accident occurs. The RL-based agents would learn from patterns in spatiotemporal traffic data, vehicle behaviors, and environmental conditions to anticipate potential hazards. By continuously adapting through feedback and interaction with the environment, predictive agents could issue timely alerts to drivers or traffic management systems, thereby reducing the likelihood of collisions. Future research will explore the design, training, and integration of such RL agents within the current multi-agent architecture, focusing on balancing prediction accuracy, system latency, and real-time applicability.

Moreover, future work will explore geometry-aware 3D point cloud learning and advanced 3D vision with dual-frequency LiDAR imaging to enhance spatial perception and scene understanding in complex traffic environments [49,50,51].

## Figures and Tables

**Table 2:** Summary of the dataset characteristics extracted from real-world traffic videos.

Attribute	Value
Total Videos	157
Total Size	4.28 GB
Frame Extraction Rate	0.5 FPS
Total Frames	∼10,000
“Accident” Frames	∼5200
“No Accident” Frames	∼4800
Annotation Tool	CVAT
Annotation Hours	215 h
Format for Training	YOLOv11-compatible

**Table 3:** Dataset splitting distribution for experimental evaluation.

Class	Training (80%)	Validation (20%)
Accident	4160	1040
No Accident	3840	960
Total	8000	2000

**Table 4:** Hardware specifications for experimental evaluation.

Component	Specification
Processor	Intel^®^ Core™ i9-13900 (13th Gen)
Memory	64 GB DDR5
GPU	Zotac^®^ GeForce RTX 4090 Trinity, 24 GB GDDR6X
Storage	1 TB NVMe PCIe Gen4 SSD

**Table 5:** Core classifier training configuration details.

Parameter	Specification
Model Architecture	Pre-trained YOLOv11
Total Epochs	500
Batch Size	32 or 64 (adaptive to GPU memory)
Optimizer Used	SGD
Initial Learning Rate	0.003
LR Scheduler	Linear Warm-up + Cosine Annealing
Framework	Ultralytics YOLOv11
Processing Hardware	CUDA-enabled GPU
Classification Task	Binary (Accident/No Accident)
Input Format	Annotated Images (YOLO format)

**Table 6:** Summary of evaluation metrics and corresponding formulas for classification performance, computational efficiency, and descriptive model characteristics.

No.	Formula	Description
**Classification Metrics**		
1	$\mathrm{Accuracy}\;\left(\%\right)=\left(\frac{TP+TN}{TP+TN+FP+FN}\right)\times 100$	Overall percentage of correct predictions.
2	$\mathrm{Precision}\;\left(\%\right)=\left(\frac{TP}{TP+FP}\right)\times 100$	Proportion of predicted accidents that are correct.
3	$\mathrm{Recall}\;\left(\%\right)=\left(\frac{TP}{TP+FN}\right)\times 100$	Proportion of actual accidents correctly identified.
4	$F1-\mathrm{Score}=2\times \left(\frac{\mathrm{Precision}\times \mathrm{Recall}}{\mathrm{Precision}+\mathrm{Recall}}\right)$	Balance between Precision and Recall.
5	$\mathrm{TPR}=\frac{TP}{TP+FN}$	True Positive Rate (sensitivity) for ROC analysis.
6	$\mathrm{FPR}=\frac{FP}{FP+TN}$	False Positive Rate for ROC analysis.
7	$\mathrm{AUC}=\int_{0}^{1}\mathrm{TPR}\left(\mathrm{FPR}\right)\;d\left(\mathrm{FPR}\right)$	Area under the ROC curve.
**Computational Metrics**		
8	$\mathrm{FPS}=\frac{\mathrm{Total}\;\mathrm{Frames}\;\mathrm{Processed}}{\mathrm{Total}\;\mathrm{Time}\;\left(\mathrm{seconds}\right)}$	Number of frames processed per second during inference.
9	$\mathrm{Parameters}\;\left(M\right)=\frac{\mathrm{Total}\;\mathrm{Trainable}\;\mathrm{Parameters}}{1,000,000}$	Total model parameters measured in millions.
**Descriptive Model Evaluation Metrics**		
10	$\mathrm{BLEU}=BP\times \exp\left(\sum_{n=1}^{N}w_{n}\log p_{n}\right)$	N-gram precision metric with brevity penalty.
11	$\mathrm{METEOR}=F_{\mathrm{mean}}\times \left(1-\gamma P_{\mathrm{frag}}\right)$	Precision–recall based metric with fragmentation penalty.
12	$\mathrm{ROUGE}-L=\frac{LCS(\mathrm{candidate},\mathrm{reference})}{\mathrm{Length}\;\mathrm{of}\;\mathrm{reference}}$	Longest common subsequence-based text similarity metric.

**Table 7:** Comprehensive classification and computation performance comparison of YOLOv11, ResNet-50, and ViT on custom dataset.

Metric	YOLOv11	ResNet-50	ViT
Classification Performance			
Accuracy (%)	86.5	83.29	85.83
Precision (%)	86.6	78.97	81.65
Recall (%)	85.5	92.43	93.77
F1-Score (%)	86.1	85.17	87.29
Computational Performance			
Inference Speed (FPS)	393	500	312.5
Number of Parameters (M)	28.34	11.18	85.80
Best Use Case	Best Precision	Fastest Inference	Highest Recall

**Table 8:** Training performance of YOLOv11, showing the evolution of learning rate, training/validation losses, and classification accuracy over epochs.

Epoch	λ	Train Loss	Accuracy (%)	Val Loss
498	$1.59\times 10^{-4}$	0.048	86.57	1.00
499	$1.39\times 10^{-4}$	0.043	86.57	1.00
500	$1.19\times 10^{-4}$	0.048	86.52	1.00

λ represents the learning rate.

**Table 9:** Confusion matrix summarizing YOLOv11’s classification results on the validation set.

	Predicted: Accident	Predicted: No Accident
Actual: Accident	TP = 828	FN = 139
Actual: No Accident	FP = 128	TN = 916
Missed Accidents	14% (139/967)	
False Alarms	12% (128/1044)	

**Table 10:** Comparison of different models using BLEU, METEOR, and ROUGE-L metrics.

Model	BLEU	METEOR	ROUGE-L
VideoLLaMA3	0.0755	0.2258	0.3625
Qwen2-VL	0.0320	0.2013	0.2359
LlavaNextVideo	0.0278	0.2302	0.2534
LlavaOnevision	0.1039	0.3186	0.3306

**Table 11:** Overall CIRS framework performance for emergency responses.

	YOLOv11	VLM	Overall
Speed (second)	3–7 ms/frame	2–4 s/video	3–5 s

## Data Availability

The dataset includes publicly available videos (links on request) and proprietary CCTV recordings from the Central Police Office, Karachi. Proprietary data are restricted for privacy reasons but may be shared by the corresponding author with official permission.
